# Clinical application of metagenomic next-generation sequencing in the diagnosis of severe pneumonia pathogens

**DOI:** 10.3389/fcimb.2025.1661213

**Published:** 2025-11-06

**Authors:** Raojuan Huang, Ying Zhang, Caitao Dong, Jingdi Chen, Handong Zou, Yang Liu, Mengmeng Guo, Hang Gao, Quan Ke, Wei Wu

**Affiliations:** 1 Renmin Hospital of Wuhan University, Wuhan, Hubei, China; 2 Department of Vascular Surgery, Renmin Hospital of Wuhan University, Wuhan, Hubei, China; 3 Department of Urology, Renmin Hospital of Wuhan University, Wuhan, Hubei, China; 4 Department of Orthopedics, the Airborne Military Hospital, Wuhan, Hubei, China; 5 Department of Critical Care Medicine, Renmin Hospital of Wuhan University, Wuhan, Hubei, China; 6 Department of Critical Care Medicine, Xianfeng County People’s Hospital, Enshi, Hubei, China

**Keywords:** severe pneumonia, metagenomic next-generation sequencing, pathogen, clinical diagnosis, intensive care unit

## Abstract

**Background:**

Severe pneumonia is a significant cause of mortality among ICU patients. Metagenomic next-generation sequencing (mNGS) is an advanced, comprehensive, unbiased diagnostic tool for pathogen identification in infectious diseases. This study aimed to evaluate the clinical efficacy of mNGS for diagnosing severe pneumonia.

**Methods:**

This study retrospectively analyzed 323 patients with suspected severe pneumonia admitted to the intensive care unit (ICU) of Wuhan University Renmin Hospital between January 2022 and December 2023. Bronchoalveolar lavage fluid (BALF) samples were collected from all 323 patients, and blood samples were obtained from 80 patients. Both mNGS and conventional microbial testing (CMT) were performed on the collected BALF and blood samples to analyze the pathogen spectrum. The diagnostic performance of mNGS and CMT was systematically evaluated and compared.

**Results:**

The overall positivity rate of mNGS was significantly greater than that of CMT (93.5% vs. 55.7%, p < 0.001). mNGS demonstrated significantly greater sensitivity than did CMT (94.74% vs. 57.24%, p < 0.001) but lower specificity (26.32% vs. 68.42%, p < 0.01). mNGS identified 36 bacterial species, 14 fungal species, 7 viral species, and 1 Chlamydia species, whereas CMT detected 21 bacterial species and 9 fungal species. According to the pathogen spectrum, Klebsiella pneumoniae, Acinetobacter baumannii, and Candida albicans were the predominant pathogens associated with severe pneumonia. The detection rate of mixed infections was significantly higher with mNGS than with CMT (62.8% vs. 18.3%, p < 0.001).

**Conclusions:**

Compared with CMT methods, mNGS has significant advantages in pathogen detection for severe pneumonia. Owing to its broad detection range and high sensitivity, mNGS serves as a valuable complementary approach to traditional culture-based methods.

## 1 Introduction

Severe pneumonia is a prevalent condition in the ICU, with mortality rates ranging from 20% to 50% (Huang et al., 2024; Xie et al., 2024). It is caused by a diverse spectrum of pathogens, including bacteria, fungi, viruses, and atypical pathogens (Decker and Forrester, 2022; Contes and Liu, 2025; Liu et al., 2025). Timely and accurate etiological diagnosis is crucial for developing effective treatment strategies, reducing the adverse effects of empirical antibiotic therapy (Vaughn et al., 2024), and ultimately improving patient prognosis. However, conventional pathogen detection methods such as microbial culture, polymerase chain reaction (PCR), and serological testing are limited by their detection speed and sensitivity (Chen and Qin, 2024), often failing to meet the diagnostic demands of severe pneumonia patients.

Metagenomic next-generation sequencing (mNGS) represents an advanced high-throughput sequencing technology that can directly sequence DNA or RNA from all pathogens in clinical samples. Unlike traditional methods, it does not require pathogen isolation or culture, making it faster and more comprehensive for identifying infections (Diao et al., 2022). Numerous studies have demonstrated that mNGS significantly enhances pathogen detection (Han et al., 2023; Xiao et al., 2023; Gao et al., 2024) and has unique advantages in identifying viruses, fungi, and atypical pathogens (He et al., 2022). Recent systematic reviews and meta-analyses have further confirmed that mNGS demonstrates higher sensitivity than conventional methods for pathogen identification (Chen et al., 2022; Yan et al., 2025). Furthermore, mNGS holds great potential for detecting unknown or rare pathogens (Bassi et al., 2022; Zhu et al., 2022). It also demonstrates high clinical applicability across a wide range of infectious diseases, including respiratory tract infections (Dong et al., 2023; Gao et al., 2024), central nervous system infections (Benoit et al., 2024), urinary tract infections (Jia et al., 2023), periprosthetic joint infections (Indelli et al., 2021), and spinal infections (Lv et al., 2024). Therefore, evaluating the clinical efficacy of mNGS is essential for optimizing diagnostic strategies and improving patient outcomes.

However, current research on the application of mNGS in ICU patients with severe pneumonia is still limited, and some drawbacks need to be urgently addressed. For example, mNGS struggles to distinguish between harmless colonizers and harmful pathogens. It is also prone to sample contamination and false positives due to sequencing errors (Jiang et al., 2023). Moreover, mNGS is still controversial due to its high costs, method standardization, and interpretation of results (He et al., 2022; Zhang P. et al., 2024). To promote the standardized application of mNGS in clinical practice, in-depth clinical research is needed. Therefore, this study aimed to evaluate and compare the clinical efficacy of mNGS and CMT in the diagnosis of pathogens causing severe pneumonia, analyze the distribution of the pathogen spectrum of severe pneumonia, and analyze the impact of different samples on the detection efficacy of mNGS in detail.

## 2 Materials and methods

### 2.1 Patient enrollment

A total of 323 patients with suspected severe pneumonia hospitalized in the ICU of Renmin Hospital of Wuhan University from January 2022 to December 2023 were retrospectively included. The diagnostic criteria for severe pneumonia referred to the official clinical practice guidelines of the Infectious Diseases Society of America/American Thoracic Society (Metlay et al., 2019). The exclusion criteria were as follows: (1) age ≤ 18 years; (2) pregnancy; and (3) incomplete case data. The study was approved by the ethics committee of Renmin Hospital of Wuhan University.

### 2.2 Sample collection

All enrolled patients underwent bronchoalveolar lavage fluid (BALF) collection via fiberoptic bronchoscopy. The BALF collection procedure was performed as follows: after local anesthesia with 2% lidocaine, a fiberoptic bronchoscope was inserted into the most severely affected lung segments or subsegments, as determined by microscopic observation and imaging examinations. The targeted segments were then lavaged with multiple aliquots of sterile saline (20–50 mL) at 37 °C. At least 40% of the instilled fluid was subsequently aspirated and collected into sterile containers using a suction device. Each patient’s BALF sample was equally divided into two portions and sent to our hospital’s Laboratory Department for mNGS and CMT testing. Additionally, blood samples were collected from 80 patients for concurrent mNGS and CMT testing.

### 2.3 MNGS

#### 2.3.1 Nucleic acid extraction and mNGS detection

Nucleic acid extraction was conducted on collected samples using QIAGEN’s QIAamp Pathogen Kit (Germany) in strict accordance with the manufacturer’s protocol, as previously described (Zhan et al., 2024). Subsequently, nucleic acid sequencing was performed using the NextSeq 550DX platform. Following sequencing completion, a rigorous quality control process was implemented, which involved the removal of low-quality reads, adapter contamination, duplicate sequences, and short reads (<36 bp) to obtain high-quality sequencing data (Yin et al., 2021). Host-derived sequences were then eliminated through alignment with the human reference genome. The remaining microbial sequences were systematically aligned against the NCBI genomic database for comprehensive microbial identification and quantification.

#### 2.3.2 Interpretation of mNGS results

Based on the description of the mNGS process outlined in a previous study (Zeng et al., 2022), the following criteria were established to define mNGS positivity: (1) For bacteria (excluding *Mycobacterium* spp., *Nocardia* spp., and *Legionella pneumophila*), fungi, and viruses, a minimum of three nonoverlapping reads specific to the detected species were needed. (2) For *Mycobacterium* spp., *Nocardia* spp., and *Legionella pneumophila*, the presence of at least one species-specific read was considered sufficient for positivity. (3) A detected read ratio to the negative template control (NTC) of less than 10 was classified as negative.

### 2.4 Statistical analysis

Statistical analyses were performed using SPSS 26.0 (IBM, USA). Continuous variables with a normal distribution were analyzed using the t test and are expressed as the mean ± standard deviation, whereas nonnormally distributed continuous variables were analyzed using the Mann-Whitney U test for independent samples or the Wilcoxon test for paired samples and are expressed as the median and interquartile range (IQR). Categorical variables were analyzed using the chi-square test; if the expected frequency was <5, we used Fisher’s exact test, and the results were expressed as frequencies and percentages. For categorical variables, the 95% confidence intervals for point estimates were calculated using the Clopper-Pearson exact method when sample sizes were small or proportions approached extreme values. Although no formal sample size calculation was performed for this retrospective study, *post-hoc* analysis confirmed adequate power (>90%) for primary outcomes. A two-tailed p value < 0.05 was considered statistically significant.

## 3 Results

### 3.1 Patient characteristics

**Figure 1:**
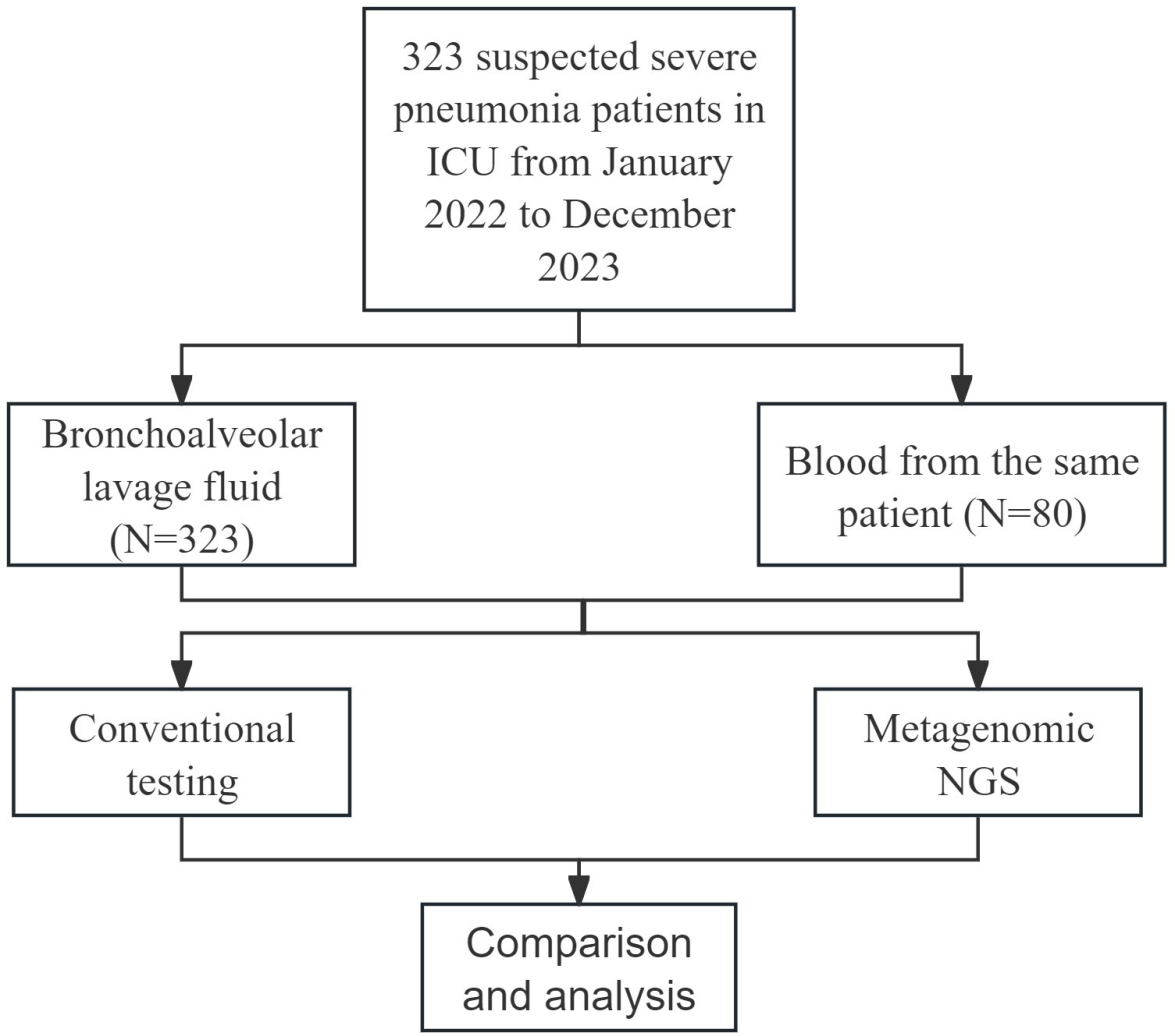
Enrollment details and study design.

As shown in **Figure 1**, a total of 323 patients were ultimately enrolled in this study. BALF samples were collected from all 323 patients, and blood samples were obtained from 80 patients. Both mNGS and CMT were performed on these samples. The clinical and demographic characteristics of the patients are presented in **Table 1**. Among the 323 patients, 237 (73.4%) were male, and 86 (26.6%) were female. The median age of the patients was 64 years, with an age range of 21 to 94 years. Underlying medical conditions were present in 212 (65.6%) patients, including 193 (59.75%) with cardiovascular diseases, 31 (9.60%) with renal diseases, 1 (0.31%) with autoimmune diseases, 58 (17.96%) with diabetes, and 70 (21.67%) with malignant tumors. During hospitalization, 296 (91.6%) patients received invasive mechanical ventilation. The 30-day mortality analysis revealed 177 deaths (54.8%).

**Table 1:** Baseline data of the included patients.

Characteristic	Level	Data
Age (median [IQR])	Range (21~94)	64.0 [53.5~72.5]
Sex, n (%)	Male	237 (73.4)
	Female	86 (26.6)
Underlying disease, n (%)	Cardiovascular and cerebrovascular diseases	193 (59.8)
	Renal diseases	31 (9.6)
	Autoimmune diseases	1 (0.3)
	Dabetes	58 (18.0)
	Malignant tumour	70 (21.7)
Invasive ventilator assisted ventilation, n (%)		296 (91.6)
30-day mortality, n (%)		177 (54.8)

### 3.2 Positive detection rates between mNGS and CMTs

As shown in **Table 2**, the overall positive detection rate of mNGS was 93.5% (302/323), which was significantly greater than that of CMT (55.7%, 180/323) (p < 0.001). Among the BALF samples from 323 patients, the positive detection rate of mNGS was 93.2% (301/323), which was significantly higher than that of CMT (52.9%, 171/323) (p < 0.001). Similarly, for blood samples from 80 patients, the positive detection rate of mNGS was 87.5% (70/80), which was also significantly higher than that of CMT (32.5%, 26/80) (p < 0.001).

**Table 2:** Comparison of positive detection rates between mNGS and CMT.

Sample type	Testing method	Positive cases	Positive rate (%)	P value
Total (n=323)	mNGS	302	93.5	<0.001
	CMT	180	55.7	
BALF (n=323)	mNGS	301	93.2	<0.001
	CMT	171	52.9	
Blood (n=80)	mNGS	70	87.5	<0.001
	CMT	26	32.5	

### 3.3 Results of pathogen detection

**Figure 2:**
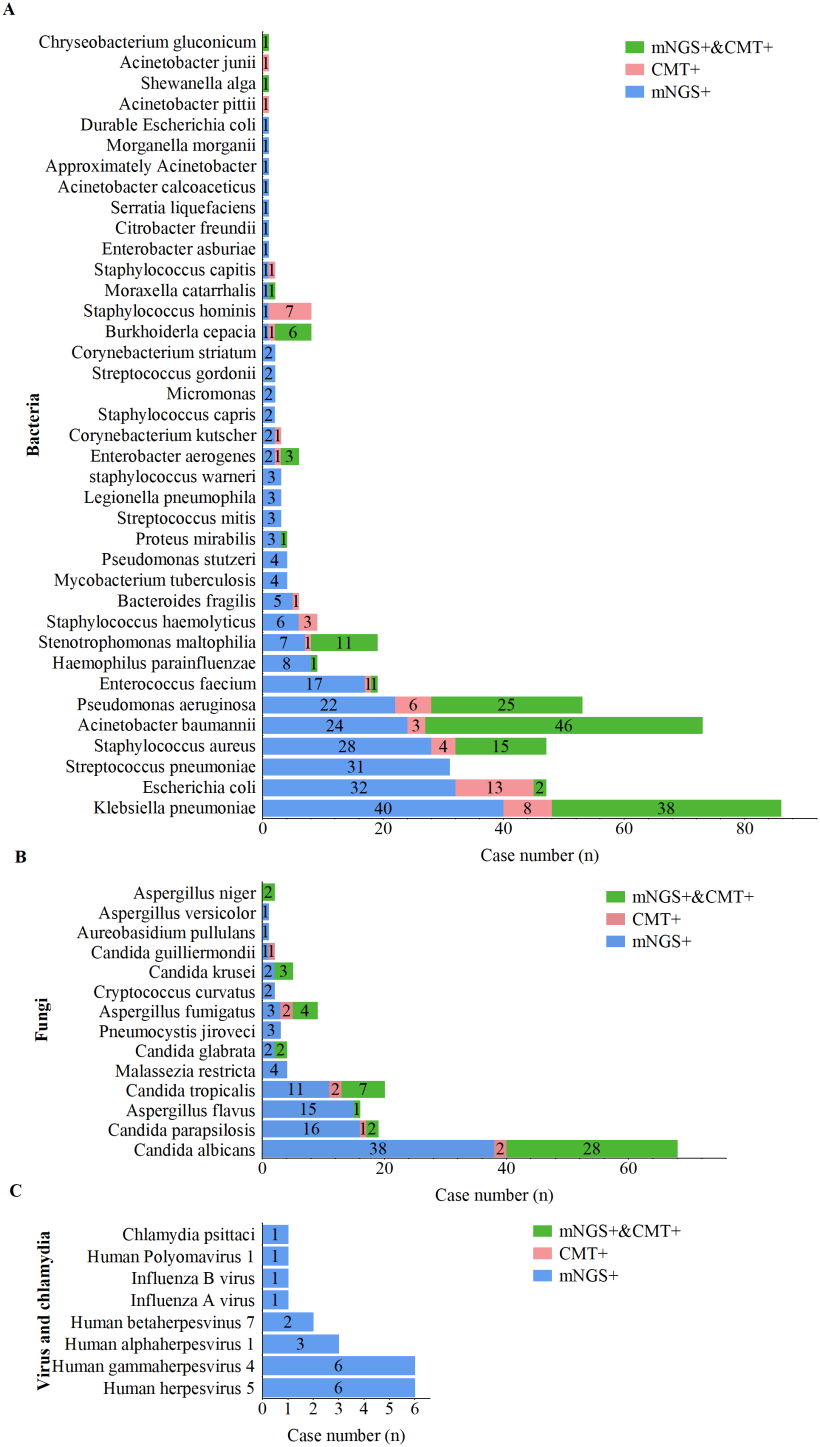
Distribution of pathogens detected by mNGS and CMT. **(A)** Bacterial pathogens detected by mNGS and CMT. **(B)** Fungal pathogens detected by mNGS and CMT. **(C)** Viral pathogens detected by mNGS and CMT.

The distribution of pathogens detected in this study is shown in **Figure 2**. A total of 56 unique pathogens were identified by mNGS and CMT, including 38 bacteria, 14 fungi, 7 viruses, and 1 Chlamydia. mNGS detected a broader spectrum of pathogens than did CMT, identifying 36 bacteria, 14 fungi, 7 viruses, and 1 Chlamydia, whereas CMT detected only 21 bacteria and 9 fungi.

**Figure 3:**
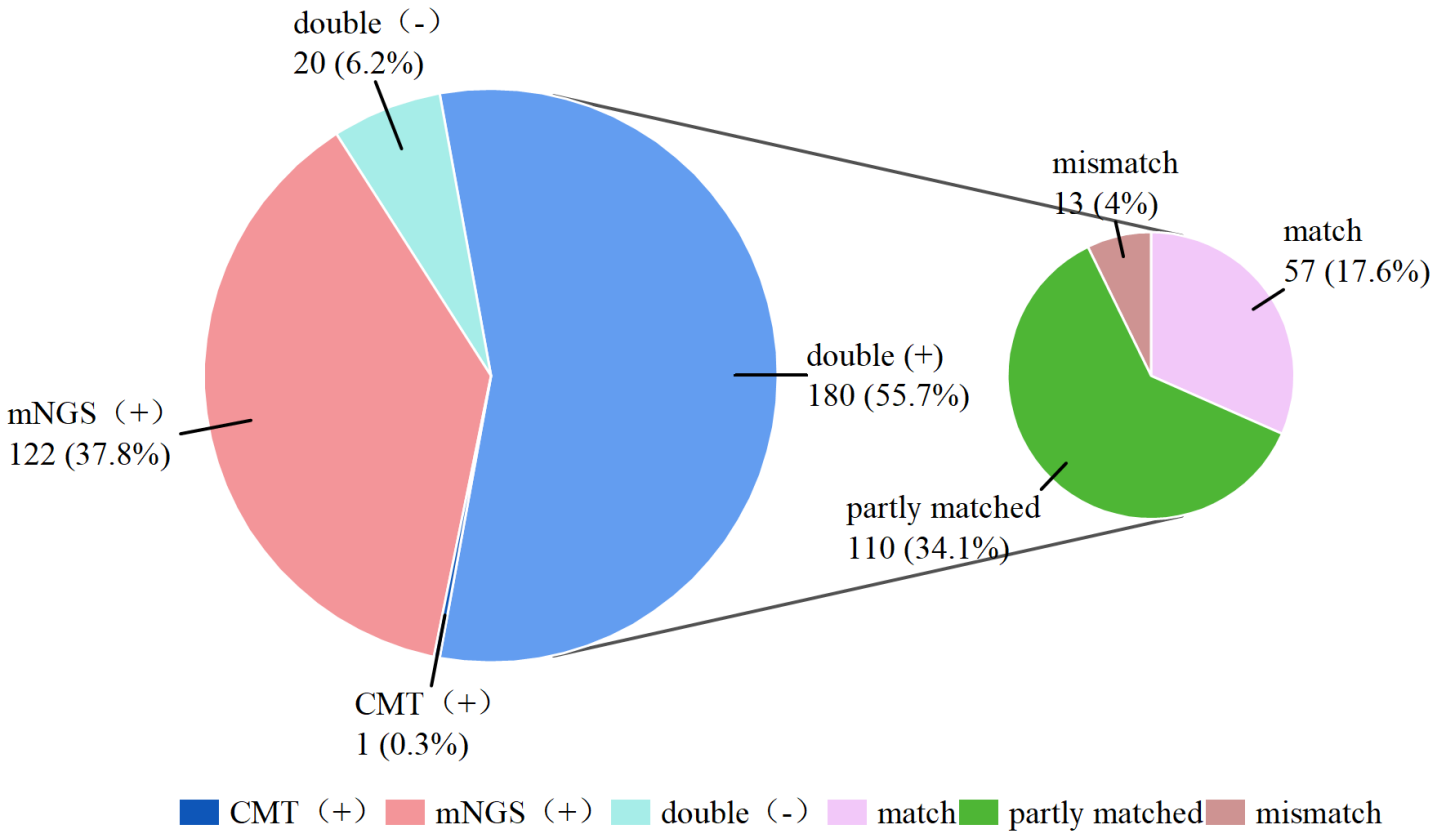
Consistency between mNGS and CMT.

For bacterial pathogens (**Figure 3A**), Klebsiella pneumoniae was the most frequently detected species by both methods, followed by Acinetobacter baumannii, Pseudomonas aeruginosa, and Staphylococcus aureus. Notably, 17 bacterial species, including Streptococcus pneumoniae, Haemophilus parainfluenzae, Enterococcus faecalis, Mycobacterium tuberculosis, and Legionella pneumophila, were detected exclusively by mNGS. The bacterial detection rate by mNGS (83.3%, 269/323) was significantly greater than that by CMT (45.2%, 146/323; p < 0.001).

For fungal pathogens (**Figure 3B**), *Candida albicans* was the most frequently detected species by both methods. All fungal pathogens identified by CMT were also detected by mNGS; however, species such as Malassezia restricta, Pneumocystis jirovecii, Cryptococcus flexneri, Aureobasidium pullulans, and Aspergillus heterocysticus were detected exclusively by mNGS. Additionally, the fungal detection rate by mNGS (44.6%, 144/323) was significantly greater than that by CMT (17.6%, 57/323; p < 0.001).

For viral and Chlamydia pathogens (**Figure 3C**), mNGS demonstrated a significant advantage, with all viruses and Chlamydia (including one case of Chlamydia psittaci) detected exclusively by mNGS. Viral profile analysis revealed that the predominant viral pathogens detected belonged to the human herpesvirus family, including cytomegalovirus, Epstein–Barr virus (EBV), HHV-1, and HHV-7.

### 3.4 Consistency of mNGS with conventional culture

As shown in **Figure 3**, 180 (55.7%) of the 323 patients were positive according to both mNGS and CMT double positive, and 20 (6.2%) patients had negative results according to both testing methods double negative, resulting in an overall concordance rate of 61.9%. Furthermore, only 1 (0.3%) patient was positive by CMT alone (negative by mNGS), whereas 122 (37.8%) patients were positive only by mNGS (negative by CMT). Among the double-positive results, 57 cases were completely matched between the mNGS and CMT assays, 110 cases were partly matched (with partially overlapping pathogens detected by both methods), and 29 cases were completely mismatched. The detailed pathogen distribution is compiled in **Supplementary Table S1**. Kappa analysis indicated slight concordance between the two methods (kappa = 0.149).

### 3.5 Diagnostic performance of the mNGS and CMT

A total of 304/323 (90.4%) patients were ultimately diagnosed with severe pneumonia, and the remaining 19 were diagnosed with noninfectious lung disease or extrapulmonary disease. The diagnostic performance of mNGS and CMT is shown in **Table 3**. Among the 323 patients with suspected severe pneumonia, mNGS yielded 288 true-positive and 5 true-negative results. Overall, mNGS had a sensitivity of 94.74% (95% CI: 91.59%-96.96%), which was significantly greater than that of CMT (57.24%, 95% CI: 51.46%-62.87%) (p < 0.001). The specificities of mNGS and CMT were 26.32% (95% CI: 9.15%-51.20%) and 68.42% (95% CI: 43.45%-87.42%), respectively, with CMT showing significantly higher specificity than mNGS (p < 0.05). The positive predictive values (PPVs) of mNGS and CMT were 95.36% (95% CI: 92.34%-97.44%) and 96.67% (95% CI: 92.89%-98.77%), respectively, whereas the negative predictive values (NPVs) were 23.81% (95% CI: 8.22%-47.17%) and 9.09% (95% CI: 4.93%-15.04%), respectively. There was no significant difference in the PPV between mNGS and CMT, but the NPV of mNGS was significantly greater than that of CMT (23.81% vs. 9.09%, p < 0.05). The mNGS assay for BALF and blood samples demonstrated higher sensitivity but lower specificity than did the CMT for the corresponding samples (p < 0.001). The turnaround time for mNGS in BALF samples was 1.92 ± 0.68 days, significantly shorter than CMT (2.30 ± 0.74 days, P < 0.001). The time-saving effect was even more pronounced in blood samples, where mNGS achieved results in 1.91 ± 0.78 days versus 4.40 ± 1.07 days with CMT (P < 0.001) (**Table 4**).

**Table 3:** Diagnostic performance of mNGS and CMT in suspected severe pneumonia.

Sample type	Diagnostic testing	Sensitivity (%) (95% CI)	Specificity (%) (95% CI)	PPV (%) (95% CI)	NPV (%) (95% CI)
Total (n=323)	mNGS	94.74 (91.59-96.96)	26.32 (9.15 - 51.20)	95.36 (92.34-97.44)	23.81 (8.22-47.17)
	CMT	57.24 (51.46-62.87)	68.42 (43.45-87.42)	96.67 (92.89-98.77)	9.09 (4.93-15.04)
BALF (n=323)	mNGS	94.41 (91.20-96.71)	26.32 (9.15-51.20)	95.35 (92.32-97.43)	22.73 (7.82-45.37)
	CMT	55.26 (49.48-60.94)	84.21 (60.42-96.62)	98.25 (94.96-99.64)	10.53 (6.14-16.53)
Blood (n=80)	mNGS	90.41 (81.24-96.06)	42.86 (9.90-81.59)	94.29 (86.01-98.42)	30.00 (6.67-65.25)
	CMT	32.88 (21.33-44.87)	71.43 (29.04-96.33)	92.31 (74.87-99.05)	9.26 (3.08-20.30)

**Table 4:** The turnabout time of mNGS and CMT.

Sample type	Diagnostic testing	Turnabout time	P value
BALF	mNGS	1.92 ± 0.68 days	<0.001
	CMT	2.30 ± 0.74 days	
Blood	mNGS	1.91 ± 0.78 days	<0.001
	CMT	4.40 ± 1.07 days	

### 3.6 The performance of mNGS and CMT in the diagnosis of single and mixed pathogen infections

**Figure 4:**
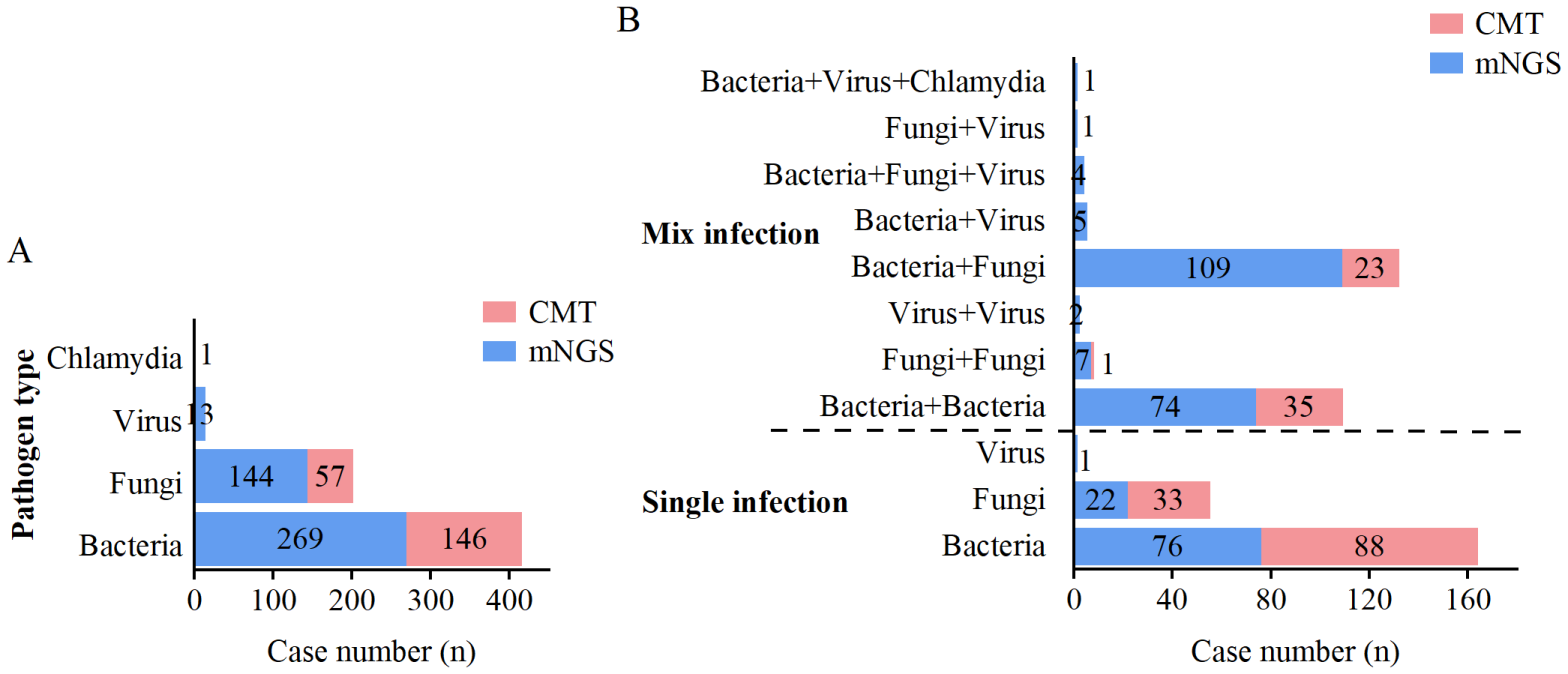
Distribution of single and mixed infections detected by mNGS and CMT. **(A)** Distribution of pathogen types detected by mNGS and CMT. **(B)** Distribution of single and mixed infection types detected by mNGS and CMT.

When two or more pathogens were detected, the result was defined as a mixed pathogen infection. As shown in **Table 5**, mNGS detected 99 (30.7%) positive cases for single pathogens and 203 (62.8%) positive cases for mixed pathogens, whereas CMT detected 121 (37.5%) positive cases for single pathogens and 59 (18.3%) positive cases for mixed pathogens. The overall positivity rate of mNGS for mixed infections was significantly greater than that of CMT (p<0.001). mNGS demonstrated superior performance to CMT in terms of species diversity and detection rates for all pathogen types, including bacteria, fungi, viruses, and chlamydia (**Figure 4A**). Among single infections, bacterial infections were the most common. In mixed infections, the most frequently detected combination by mNGS was bacterial-fungal (109/323, 33.7%), followed by bacterial-bacterial (74/323, 22.9%). In contrast, CMT detected only 23 (7.1%) bacterial-fungal infections, 35 (10.8%) bacterial-bacterial infections, and 1 (0.3%) fungal-fungal mixed infection. Notably, mixed infections involving viruses were detected exclusively by mNGS. mNGS identified a significantly greater number of mixed infection types than did CMT (**Figure 4B**). In cases of mixed infections, mNGS failed to identify 5 cases of Pseudomonas aeruginosa, 5 cases of Klebsiella pneumoniae, 2 cases of Staphylococcus aureus, 2 cases of Candida tropicalis, and 1 case of Candida albicans, whereas CMT was positive.

**Table 5:** Comparison of single and mixed pathogen infections detected by mNGS and CMT.

Infection type	mNGS, n (%)	CMT, n (%)	P value
Single Pathogen	99 (30.7%)	121 (37.5%)	0.049
Mixed Pathogens	203 (62.8%)	59 (18.3%)	< 0.001
Total Positive	302 (93.5%)	180 (55.7%)	< 0.001

## 4 Discussion

Severe pneumonia is associated with high mortality and pulmonary and extrapulmonary complications (Niederman, 2022; Salluh et al., 2024). The lack of effective early pathogenetic diagnosis and treatment in patients with severe pneumonia may lead to disease progression with complications such as life-threatening sepsis and multiorgan failure, further increasing the risk of mortality (Collaborators, 2022). Pathogenic culture is the gold standard for the clinical diagnosis of infectious diseases. However, early empirical antibiotic treatment, slow growth of pathogen cultures, and strict requirements for the culture environment reduce the sensitivity of traditional culture methods (Jia et al., 2023). mNGS, a highly efficient and unbiased technology that is not dependent on culture, can potentially overcome the limitations of traditional detection methods and provide valuable pathogenic diagnostic tools for infectious diseases.

Among the 323 patients with suspected severe pneumonia included in this study, mNGS exhibited significantly higher overall positivity rates than CMT (93.5% vs. 55.7%), and this superiority was maintained across both BALF and blood specimens, which was in line with the findings of Jiang et al (Jiang et al., 2024).

A comparison of the diagnostic performance of mNGS and CMT revealed that the overall sensitivity of mNGS was as high as 94.74%, which was significantly greater than that of CMT (57.24%), in agreement with the findings of a previous study (Zheng et al., 2024). It is important to note, however, that this observed difference may have been influenced by the lack of standardized timing between antibiotic administration and sample collection. As shown by Sizhou Feng et al., prior antibiotic exposure considerably impairs the performance of CMT, while exerting minimal influence on mNGS-potentially accentuating the sensitivity advantage of the latter (Feng et al., 2024). In contrast, in our research, mNGS exhibited notably lower specificity than CMT (26.32% vs. 68.42%). Although some studies have reported higher specificity for mNGS (Jin et al., 2022; Yan et al., 2025), our findings are consistent with several studies that have demonstrated a relative reduction in the specificity of mNGS (Xie et al., 2021; Zheng et al., 2024). These divergent findings are likely attributable to heterogeneity in patient cohorts, specimen sources, and laboratory methodologies among the studies. When applied in the ICU setting, the superior sensitivity of mNGS increases the detection of environmental contaminants, respiratory colonizers, and non-viable pathogen fragments, consequently leading to more false-positive findings than CMT. Furthermore, the use of a fixed read-count threshold for determining positivity, though clinically practical and widely adopted (Zeng et al., 2022), is inherently more prone to these false-positive signals than more refined, normalized metrics such as Reads Per Million ratio (RPM-r) (Miller et al., 2019). To maximize clinical utility and minimize misinterpretation, a structured framework for interpreting mNGS results was proposed (Elbehiry and Abalkhail, 2025). First, all positive mNGS results must be strictly correlated with definitive clinical evidence of infection, such as progressive pulmonary infiltrates on imaging and elevated inflammatory markers like procalcitonin (PCT) and C-reactive protein (CRP). Second, diagnostic weight should be assigned according to the pathogen: high-concern pathogens (e.g., Mycobacterium tuberculosis, Legionella pneumophila) should be prioritized over common colonizers (e.g., oral streptococci). Finally, establishing laboratory-validated, pathogen-specific thresholds constitutes a critical goal for future research.

In terms of sample selection, some studies have suggested testing both BALF and blood samples from patients with severe pneumonia because pathogens in these patients are likely to enter the bloodstream from the lungs, and performing a blood mNGS test can partially predict the presence of pathogens in BALF (Chen et al., 2021). Our results revealed that the positive rate, sensitivity, specificity, and NPV of the BALF mNGS test were greater than those of blood mNGS. Therefore, we suggest that BALF samples should be prioritized for mNGS testing in the diagnosis of pathogens in patients with severe pneumonia, and whether to use blood mNGS testing should be decided on the basis of the economic cost and complexity of the infection. Regarding the cost-effectiveness of mNGS, current research indicates that mNGS accounts for 30-50% of total microbiology testing costs, with a single mNGS test being significantly more expensive than conventional methods (approximately $2,000-$2,900) (Zhang H. et al., 2024). However, these higher costs may potentially be offset by factors such as reduced time to pathogen identification and decreased unnecessary antibiotic use. Future studies are needed to better weigh the diagnostic benefits of early mNGS testing against its economic burden. The turnaround time for mNGS was significantly shorter than CMT (BALF: 1.92 ± 0.68 days vs 2.30 ± 0.74 days; blood: 1.91 ± 0.78 days vs 4.40 ± 1.07 days; P<0.001 for both comparisons). This reduction in diagnostic time provides a critical window for early targeted antimicrobial therapy in severe pneumonia management.

We further analyzed the distribution of pathogenic microorganisms in patients with severe pneumonia. The results revealed that the positivity rate of mNGS for the detection of bacterial, fungal, and viral pathogens was much higher than that of CMT. Specifically, Klebsiella pneumoniae was the bacterial pathogen most frequently detected by both methods, followed by Acinetobacter baumannii, Pseudomonas aeruginosa, Escherichia coli, and Staphylococcus aureus. These findings align with previous reports (Shean et al., 2024), as all these pathogens are often present in community- or nosocomial-based infections (Hu et al., 2022). In the present study, Candida spp. were the most common fungal pathogens, followed by Aspergillus spp. This finding is in agreement with what has been reported in previous studies (Wei et al., 2024). Although mNGS shows markedly higher sensitivity than conventional methods for detecting Candida and Aspergillus, the high frequency of respiratory colonization by these fungi makes it challenging to differentiate true infection from colonization (Meena, 2022; Shajiei et al., 2022). Consequently, the high detection rates of mNGS could overestimate the true burden of fungal disease, underscoring the need to integrate ancillary tests like galactomannan or β-D-glucan assays for accurate interpretation. For viral detection, mNGS also demonstrates superior performance compared to CMT, aligning with reports by Wang et al (Wang et al., 2023). Detection data identified human herpesviruses-notably cytomegalovirus (CMV) and Epstein-Barr virus (EBV)-as the most prevalent viral pathogens, with influenza viruses being the next most common. In patients with severe pneumonia, detection of these viruses often correlates with impaired immunity and viral reactivation (Gatto et al., 2022; Huang et al., 2023; Febbo and Revels, 2024). In critically ill patients, detecting these viruses necessitates distinguishing active disease from reactivation, which relies on assessing viral load kinetics and overall immune status. mNGS provides particular value for detecting fastidious and atypical pathogens. Pathogens such as Pneumocystis jirovecii, Mycobacterium tuberculosis, Legionella pneumophila, and Chlamydia psittaci, which are difficult to culture or identify by conventional means, are readily detectable by mNGS (Jin et al., 2022; Qu et al., 2022; Hao et al., 2023; Han et al., 2024). This capability not only confirms the utility of mNGS for diagnosing uncommon infections but also indicates that their incidence is likely underestimated in standard practice (Shi et al., 2024).

Severe pneumonia often involves polymicrobial infections. Our study confirms that mNGS identifies mixed infections at significantly higher rates than CMT, with bacteria–fungi and bacteria–bacteria co-infections being most common, consistent with prior findings (Wen et al., 2023). While this comprehensive detection highlights the value of mNGS in revealing full pathogen profiles, it also necessitates careful interpretation to distinguish true co-infections from colonization. When multiple organisms are detected, clinical assessment should prioritize primary pathogens based on quantitative reads, pathogenic potential, and clinical context (Gu et al., 2019). For example, in an immunocompetent patient with bacterial pneumonia, the presence of Aspergillus with low read counts alongside high reads of Klebsiella pneumoniae more likely reflects colonization rather than true co-infection. Notably, we observed several cases where mNGS failed to detect pathogens identified by CMT in mixed infections, including five cases of Pseudomonas aeruginosa, five of Klebsiella pneumoniae, two of Staphylococcus aureus, two of Candida tropicalis, and one of Candida albicans. These false negatives may stem from pathogen loads below the mNGS detection limit, though technical factors such as sample storage or nucleic acid degradation cannot be ruled out.

Our study has several limitations. Its single-center, retrospective design may introduce selection bias. The variable timing between empirical antibiotic administration and sample collection represents a key confounder. Additionally, specificity estimates, particularly for blood samples, show wide confidence intervals due to limited sample size. While our findings demonstrate the diagnostic potential of mNGS, they require validation through prospective, multicenter studies with protocol-defined sampling before antibiotic initiation. Future research should also investigate how mNGS-guided management affects clinical outcomes.

## 5 Conclusion

In conclusion, mNGS demonstrates high sensitivity and broad pathogen coverage for severe pneumonia, especially for atypical pathogens. However, limitations like suboptimal specificity and potential missed detection of low-abundance pathogens prevent it from fully replacing traditional methods (CMT). Combining both approaches improves diagnostic accuracy and comprehensiveness.

## Data Availability

The original contributions presented in the study are included in the article/**Supplementary Material**. Further inquiries can be directed to the corresponding author.

## Supplementary material

Supplementary Table 1 Detailed pathogen distribution data.
